# Supplementary material for: Further evidence in support of the validity of the post-secondary student stressors index using a nationwide, cross-sectional sample of Canadian university students
Source: PLoS One. 2022 Dec 15;17(12):e0278897. doi: 10.1371/journal.pone.0278897 (PMC9754283; doi:10.1371/journal.pone.0278897)
Supplement: Attachment — Submitted filename: Response to Reviewers.docx [file pone.0278897.s001.docx]

**Response to Reviewers**

Manuscript #PONE-D-22-06413: “Further evidence in support of the validity of the Post-Secondary Student Stressors Index using a nationwide, cross-sectional sample of Canadian university students”

Thank you to the reviewers for the opportunity to revise this manuscript. Please find below point-by-point responses to your inquiries, comments, and suggestions.

**Journal Requirements:**

**Comment 1:** Please ensure that your manuscript meets PLOS ONE's style requirements, including those for file naming.

**Response:** We have now confirmed our manuscript adheres to the style requirements.

**Comment 2:** Please provide additional details regarding participant consent. In the ethics statement in the Methods and online submission information, please ensure that you have specified what type you obtained (for instance, written or verbal, and if verbal, how it was documented and witnessed). If your study included minors, state whether you obtained consent from parents or guardians. If the need for consent was waived by the ethics committee, please include this information.

**Response:** We have now updated our consent statement for clarity: “Participants were provided with a letter of information and indicated their consent by selecting “I have read and understood the Letter of Information and agree to participate in this study” before being granted access to the survey questions.” (lines 89-90). This constitutes written consent. The study did not include minors.

**Comment 3:** Please confirm that [your CI statement] does not alter your adherence to all PLOS ONE policies on sharing data and materials, by including the following statement: ""This does not alter our adherence to  PLOS ONE policies on sharing data and materials.”

**Response:** Thank you - we would like to update our competing interests statement as follows:

“I have read the journal's policy and the authors of this manuscript have the following competing interests: BL is the developer of the Post-Secondary Student Stressors Index (a non-financial conflict of interest). HS is the Bell Canada Mental Health and Anti-Stigma Research Chair. This does not alter our adherence to PLOS ONE policies on sharing data and materials”

**Comments 4-5 (summarized):** We note that you have indicated that data from this study are available upon request. PLOS only allows data to be available upon request if there are legal or ethical restrictions on sharing data publicly. […] In your revised cover letter, please address the following prompt: a) If there are ethical or legal restrictions on sharing a de-identified data set, please explain them in detail (e.g., data contain potentially sensitive information, data are owned by a third-party organization, etc.) and who has imposed them (e.g., an ethics committee). Please also provide contact information for a data access committee, ethics committee, or other institutional body to which data requests may be sent.

**Response:** There are ethical restrictions to sharing this data, put in place by the ethics review board(s) from which we received clearance. This study also poses an additional level of challenge associated with data sharing as a result of having been reviewed and approved by fifteen individual post-secondary institutional research ethics boards across the country. A stipulation of our ethics approvals across all sites was that the students’ data would not be shared without an additional layer of ethics approval. Any time a secondary analysis is conducted on this data by someone other than the principal investigator for the study (author BL) - who is named data custodian across all ethics approvals – a subsequent ethics request must go through the Queen’s University’s Health Sciences and Affiliated Teaching Hospitals Research Ethics Board (HSREB) as the primary ethics body that approved this cross-Canada, multi-site study.

Although participants’ are not identified outright (i.e., by name, student number, home address, e-mail address, etc.), enough demographic information is collected that it may be possible to identify an individual participant. The data shared through this study may be sensitive in the event that a participant were to be identified (i.e., psychological distress scores, disclosure of pre-existing mental illness, etc.)

Requests regarding access to the data used in this study can be directed to the Queen’s University’s Health Sciences and Affiliated Teaching Hospitals Research Ethics Board (HSREB), though I feel it is important to note that the REB will forward that request to the named data custodian (myself -author BL). We are more than happy to share the data with other investigators, and encourage collaborative analyses! However, Queen’s does not have an established point of contact who fields external requests to investigators’ data – the data custodian is appointed to fulfill this role.

**Comment 6:** We note that you have included the phrase “data not shown” in your manuscript. Unfortunately, this does not meet our data sharing requirements […] we ask that you remove the phrase that refers to these data.

**Response:** This statement and sentence has been removed as the analyses referenced were not a core part of the research being presented.

**Comment 7:** Your ethics statement should only appear in the Methods section of your manuscript. If your ethics statement is written in any section besides the Methods, please delete it from any other section.

**Response:** We can confirm that our ethics statement appears only at the bottom of the Methods section.

**Reviewer #1:**

This manuscript is a continuation of previous studies that provides additional evidence to support the use of an indicator on stressors in students in Canada. With this, it seeks to identify the main sources of stressors and improve mental health services. The creation of the instrument and its validation fills a gap well exposed by the researchers related to the fact that there is no such tool with the participatory approach that is proposed.

**Comment 1:** Do the participating institutions have the same characteristics? I am referring to whether there may be different sources of stress depending on the profile of the participating institution. Perhaps it would help to mention that the type of institutions was comparable if they were.

**Response:** Thank you for this question. In fact, this was one of the questions we sought to answer through this cross-Canada, multi-site study, and indeed something that we are investigating in additional analyses. For example, we were interested in learning whether patterns of stress would appear to be different based on region in Canada, size of campus, and nature of campus (with respect to them being “commuter” campuses, where students typically live off campus and drive in to attend school vs. non-commuter campuses, where the vast majority of students live on or very near campus). These are all research questions that are being answered in additional sub-studies of this data, as they are beyond the scope of this particular paper.

For the purposes of this paper, we sought to investigate whether the psychometric properties of the index would remain stable and similar to what we observed in our pilot study at a single, Eastern Ontario university. In fact, we note that a major goal of the study was to collect further evidence of validity using a larger, more varied sample (line 66-68) – therefore the strong psychometrics our analyses revealed using this sample given the hypothesized variation in institutions’ characteristics lends additional strength to our findings.

**Comment 2:** Is there a difference between post-secondary education and higher education? I understand that it includes not only universities, but also other types of educational institutions. I recommend clarifying what you mean by post-secondary education.

**Response:** No, “post-secondary education” and “higher education” are synonymous. The PSSI was developed and intended to be used across different types of higher education institutions, including universities and colleges (hence “post-secondary stressors index”, and not “university stressors index”). However, the reviewer has likely noted that the majority of the participating institutions in this sample were universities (the one exception being Aurora College). Admittedly, this is a limitation of the tool as it has not yet been widely tested among college populations (this is in the works).

In response to this comment, we have now reviewed all instances throughout the manuscript where we have referred to “post-secondary” and adjusted this to “university” as appropriate (note that the word university is used when we refer to our sample or the applicability of the PSSI, but when referencing general mental health statistics for post-secondary student populations, for example, the word post-secondary is used as these stats apply to both university and college populations. We have also added a statement in our limitations section regarding the lack of testing to date among college populations potentially limiting the generalizability of the PSSI in that context (lines 283-286).

**Comment 3:** According to the domains of stressors and their respective items, (although the development of the instrument should be in another publication) could you describe how these were chosen and how was this a participatory process?

**Response:** The reviewer is correct that details regarding the development of the PSSI have been published elsewhere (please see Linden & Stuart 2019 [doi: 10.1186/s12889-019-7472-z] and Linden, Boyes & Stuart 2020 [doi: 10.1080/07448481.2020.1754222]). Briefly, the PSSI was developed through detailed consultation with students, including the selection and naming of the domains. Naming of the domains was decided over the course of multiple focus group interviews with diverse groups of students.

**Comment 4:** I recommend getting more out of the results in the discussion. An important topic that could be included in the discussion is that when identifying the sources of stress, it comes to light that most of them have nothing to do with the quality and quantity of mental health services in institutions. Rather, the sources of stress come from, for example, scheduling, organization and evaluation methods, communication and relationship between teachers and students, or even sexual harassment on campus. How do the authors think these sources of stress can be addressed on campus? can they be addressed? is there an example of that?

**Response:** With respect to this component of the author’s statement: “…when identifying the sources of stress, it comes to light that most of them have nothing to do with the quality and quantity of mental health services in institutions”, we are unable to comment as we did not do any analytic work that might provide support for a relationship (or lack of relationship) between he quality/quantity of mental health services provided on campuses and the stressors students are experiencing. While we appreciate the reviewer’s recommendation to add more discussion around the specific items that students identified as impactful sources of stress, we feel that would be beyond the scope of the present paper, which aims only to provide a concise assessment of the instrument’s psychometric properties. In fact, we have recently published an alternative paper that goes into detail regarding this exact question, exploring the stressors identified by students as impactful across each of the domains, as well as looking at whether trends appeared over time. We invite the reviewer to refer to Linden, Stuart, and Ecclestone 2022 (doi: 10.1177/07067437221111365).

**Comment 5:** I recommend changing the post-secondary keyword since it's in the title.

**Response:** This keyword has now been changed to “university”.

**Comment 6:** There is an error in the results since it says that the ages range from 16 to 20 years and in the table it is up to 30.

**Response:** If the reviewer is referring to lines 167-168 in the “Sample” subsection under results, we state that the majority of respondents fell between the ages of 16 and 20, not all of the respondents (41.4% of our sample was under the age of 21, while the other age categories account for considerable smaller proportions of the sample). For clarity we have adjusted this sentence to read “Most participants were aged 20 years or younger…” to better align with the age categories as displayed in Table 1. Please note that in reviewing this paragraph, we did notice some inconsistencies in the proportions reported in text - these have all now been fixed to align with the numbers in the table (lines 164-169).
