# Supplementary material for: Further evidence in support of the validity of the post-secondary student stressors index using a nationwide, cross-sectional sample of Canadian university students
Source: PLoS One. 2022 Dec 15;17(12):e0278897. doi: 10.1371/journal.pone.0278897 (PMC9754283; doi:10.1371/journal.pone.0278897)
Supplement: Attachment — Submitted filename: Response to Reviewers.docx [file pone.0278897.s002.docx]

**Response to Reviewers**

Manuscript #PONE-D-22-06413: “Further evidence in support of the validity of the Post-Secondary Student Stressors Index using a nationwide, cross-sectional sample of Canadian university students”

Thank you to the reviewers for the opportunity to revise this manuscript. Please find below point-by-point responses to your inquiries, comments, and suggestions.

**Journal Requirements:**

**Comment 1:** Please review your reference list to ensure that it is complete and correct. If you have cited papers that have been retracted, please include the rationale for doing so in the manuscript text, or remove these references and replace them with relevant current references. Any changes to the reference list should be mentioned in the rebuttal letter that accompanies your revised manuscript. If you need to cite a retracted article, indicate the article’s retracted status in the References list and also include a citation and full reference for the retraction notice.

**Response**: The reference list (and in-text citations) have now been reviewed and are correct.

**Reviewer Comments:**

**Comment 1:** The Abstract should be written in one paragraph only.

**Response:** We have now shortened the abstract to be the length of a single paragraph.

**Comment** **2:** The demographic data of the samples should be mentioned in the Research method, not the Research results.

**Response:** We have elected not to make this requested change as we feel it is more appropriate to have the demographic statistical results reported in the Results section with all other results.
